# 285. Post-COVID-19 Functional Status (PCFS) scale in Mexican adults: a cross-sectional study

**DOI:** 10.1093/ofid/ofac492.363

**Published:** 2022-12-15

**Authors:** Lucia Martinez Hernandez, Tania Vargas-Vazquez, Lorena Lorena Guerrero-Torres

**Affiliations:** Hospital Español, Mexico City, Distrito Federal, Mexico; Instituto Nacional De Pediatria, Mexico City, Distrito Federal, Mexico; Instituto Nacional de Nutricion SalvadorZubiran, Mexico City, Distrito Federal, Mexico

## Abstract

**Background:**

Over 5.74 million COVID-19 cases have been reported in Mexico as of May 1, 2022. Determining the impact of COVID-19 on functional status is essential to assess health services needs. The Post-COVID-19 Functional Status Scale (PCFS) is a patient-reported tool that evaluates functional status over time after COVID-19, listing a wide range of functional limitations. We aimed to assess the functional status of Mexican patients after recovery from COVID-19.

**Figure 1.**
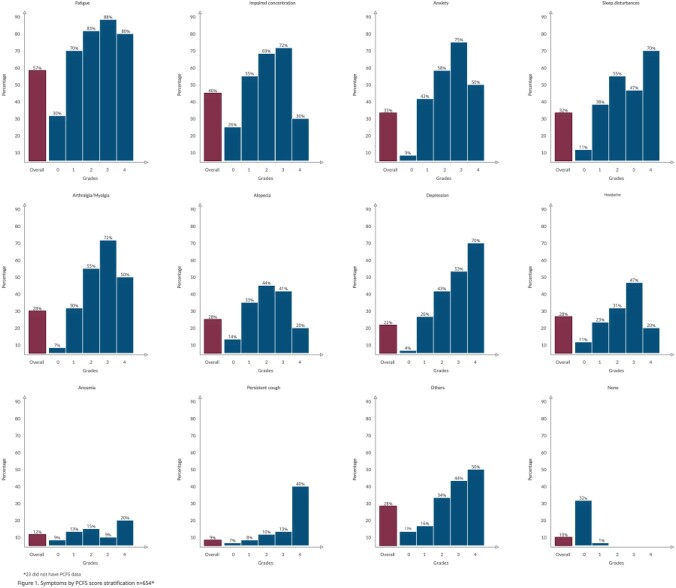
Persistent Symptoms acording the PCFS scale.

**Methods:**

This cross-sectional study was conducted from April 11 to May 1, 2022, through an online survey that included demographic and clinical data, PCFS grades 0-4 and persistent symptoms. We included Mexicans aged ≥18 years who recovered from COVID-19 (≥14 days since diagnosis), who resided in Mexico and agreed to participate. We posted the survey on Facebook and Twitter. We performed a descriptive analysis of demographic and clinical variables and assessed the relationship between symptoms and PCFS grade. Statistical analyses were conducted in Stata/SE 17.0.

**Results:**

Of 959 respondents, 838 (87%) met inclusion and exclusion criteria. Mean age was 40 (IQR 34-46) years, 82% were female, and 40% lived in Mexico City. COVID-19 diagnosis ranged from March 21, 2020, to April 7, 2022, and mean days since diagnosis was 276 (IQR 91-480) days. Most (796, 95%) were treated as outpatients. In PCFS, 338 (40%) had no functional limitations (grade 0), 266 (32%) had negligible functional limitations (grade 1), 154 (18%) had slight functional limitations (grade 2), 44 (5%) had moderate functional limitations (grade 3), 12 (2%) had severe functional limitations (grade 4) and 24 (3%) had missing PCFS data. Overall, 654 (78%) completed the persistent symptoms section. Among them, 57% presented fatigue, 46% impaired concentration, and 10% were asymptomatic (Figure 1). Of those with >12 weeks since diagnosis, 88% (499/564) had at least one symptom.

**Conclusion:**

Although most participants had mild COVID-19 at diagnosis, we found a high prevalence of persistent symptoms (90%) and functional limitations (60%) after recovery, revealing the devastating impact of COVID-19 on quality of life. The PCFS may be a valuable and simple tool to screen patients who could benefit from referral to rehabilitation programs, particularly in resource-limited settings.

**Disclosures:**

**All Authors**: No reported disclosures.

